# Cytokines as Biomarkers and Their Respective Clinical Cutoff Levels

**DOI:** 10.1155/2017/4309485

**Published:** 2017-04-09

**Authors:** Rebecca N. Monastero, Srinivas Pentyala

**Affiliations:** ^1^Department of Anesthesiology, Stony Brook University, Stony Brook, NY, USA; ^2^Department of Urology, Stony Brook University, Stony Brook, NY, USA; ^3^Department of Health Sciences, Stony Brook University, Stony Brook, NY, USA; ^4^Department of Physiology, Stony Brook University, Stony Brook, NY, USA

## Abstract

Cytokines, including interleukins, interferons, tumor necrosis factors, and chemokines, have a variety of pro- and anti-inflammatory effects in the body through a number of biochemical pathways and interactions. Stimuli, actions, interactions, and downstream effects of cytokines have been investigated in more depth in recent years, and clinical research has also been conducted to implicate cytokines in causal patterns in certain diseases. However, particular cutoffs of cytokines as biomarkers for disease processes have not been well studied, and this warrants future work to potentially improve diagnoses for diseases with inflammatory markers. A limited number of studies in this area are reviewed, considering diseases correlated with abnormal cytokine profiles, as well as specific cutoffs at which cytokines have been deemed clinically useful for diagnosing those diseases through Receiver Operator Characteristics modeling. In light of studies such as those discussed in this review, cytokine testing has the potential to support diagnosis due to its lack of invasiveness and low cost, compared to other common types of testing for infections and inflammatory diseases.

## 1. Introduction

*Cytokines and Their Role in Human Biology and Disease Processes*. Cytokines are small, nonstructural proteins, including interleukins, chemokines, interferons, and tumor necrosis factors, which have a multitude of pleiotropic effects in various organs [1]. They are released in a number of paracrine, autocrine, or endocrine pathways and have been implicated in a variety of infections and immune system-affecting disorders by both proinflammatory and anti-inflammatory mechanisms. Cytokines which have proinflammatory effects include interferon- (IFN-) *γ*, interleukin- (IL-) 17, IL-1*β*, and tumor necrosis factor- (TNF-) *α* [2, 3], and those with anti-inflammatory effects include IL-10, IL-4, and IL-1ra [2, 4]. However, the distinction between pro- and anti-inflammatory cytokine effects is not always entirely clear: pathway interactions play a major role, as individual and a combination of several cytokines can contribute to upregulation or downregulation of other cytokines and certain cytokines can have both pro- and anti-inflammatory effects [5].

Two of the most important cytokine effector pathways are the JAK-STAT and NF-*κ*B pathways [6]. These pathways are activated by cytokine ligands and are also regulated by and stimulate further release of cytokines. When a cytokine binds to a receptor in the JAK-STAT pathway, the receptor dimerizes and JAKs are activated. The JAK proteins then phosphorylate and activate the receptor as well as the STATs which are now associated with the activated receptor. This allows the STATs to dimerize and travel to the nucleus to regulate gene expression [7]. In the canonical NF-*κ*B pathway, I*κ*B proteins inhibit the *κ*B protein, in the absence of ligand, preventing *κ*B from activating transcription of genes involved in inflammation and stress responses. When cytokines are present, they serve as ligands to bind to and activate IKK complexes, which then phosphorylate I*κ*B proteins, targeting them for degradation and therefore freeing NF-*κ*B transcription factors to locate to the nucleus [8].

**Figure 1:**
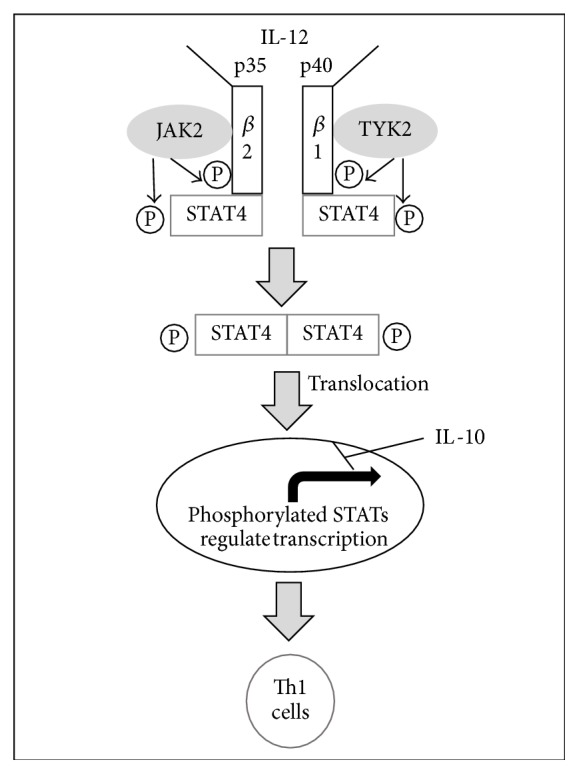
IL-12 activation of JAK-STAT pathway. IL-12 activates a series of phosphorylation events which permit STAT4 molecules to dimerize and translocate to the nucleus, upregulating a proinflammatory cascade. IL-10 has an inhibitory effect on this response transcriptionally.

**Figure 2:**
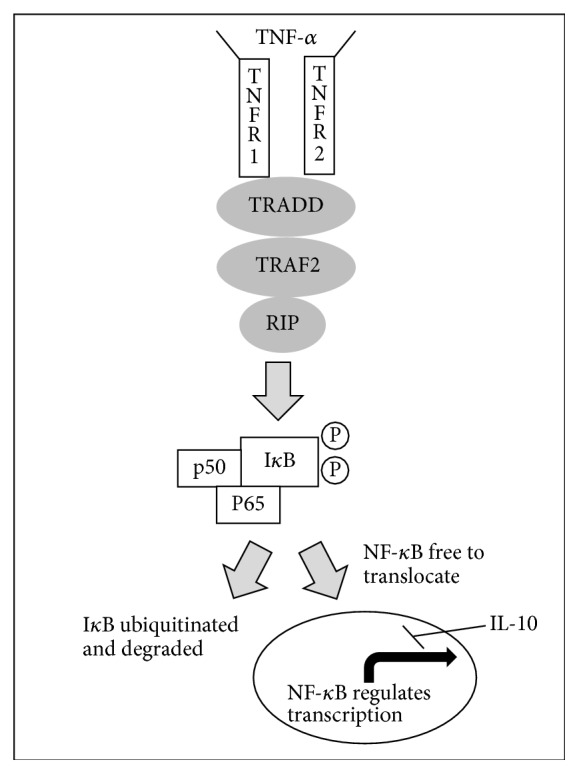
TNF-*α* activation of NF-*κ*B pathway. Upon binding with the receptor, TNF-*α* permits activation of IKK complexes, which then phosphorylate I*κ*B. Phosphorylated I*κ*B is then targeted for degradation, allowing NF-*κ*B to translocate to the nucleus to regulate tumorigenic activity and immune defense. IL-10 inhibits this response transcriptionally.

However, these pathways differ upon interaction of cytokines. When IL-12 is a ligand for the JAK-STAT pathway in macrophages, a proinflammatory response of Th1 is produced, but when IL-10 is present, IL-12's proinflammatory effects are downregulated (Figure 1). IL-10 has similar effects on other proinflammatory cytokines, reducing or even terminating their inflammatory responses [9]. In studies of TNF-*α* as a ligand in mast cells for the NF-*κ*B pathway, the result is a proinflammatory cascade [9]. Yet when that same ligand is present along with IL-10 in the pathway, this becomes muted most likely due to inhibition of NF-*κ*B, yielding a smaller inflammatory response (Figure 2). Similar to IL-10, IL-6 also is involved in various pathways which alter, or are altered by, other cytokines. IL-6 helps upregulate IL-21 for improved activity of CD4+ cells, suppresses IFN-*γ* signaling to aid in T-cell differentiation, and regulates TGF-*β* mediation of CD4+ differentiation, demonstrating the ability of multiple cytokines to differentially regulate a single pathway [10].

Further, differences in types of signaling in these pathways can also affect whether the cytokines behave in a proinflammatory or anti-inflammatory matter. IL-6 is typically thought of as proinflammatory, though it can also have anti-inflammatory effects depending on how its receptors and signaling receptor proteins interact. IL-6 signaling in the JAK-STAT pathway can behave proinflammatorily when the signaling receptor protein and IL-6 receptor are located on the same cell (classic signaling), yet the same cytokine behaves anti-inflammatorily when IL-6 binds to a soluble IL-6 receptor which activates the membrane-bound signaling receptor protein (transactivation) [10]. In a related way, IL-10 is most often thought of as an anti-inflammatory cytokine, though depending on its target cell and concentration, it can also act to promote inflammation. Activation of certain STAT proteins (STAT3 in the JAK-STAT pathway, which has anti-inflammatory effects) and inhibition of NF-*κ*B (through suppression of IKK complexes) by IL-10 play a role in inhibiting the immune response [11]. However, IL-10 has also been found to exert proinflammatory effects, stimulating immune cells including B cells and cytotoxic T cells, in high concentration [9].

Due to their vast pro- and anti-inflammatory effects, cytokines have been implicated in various disease processes. However, it is often difficult to use cytokines as diagnostic tools, although recent studies have investigated cytokine clinical diagnostic cutoffs. The limited amount of work in this area warrants future investigation to confirm diagnostic guidelines and explore guidelines for not-yet-studied cytokine-disease associations.

## 2. The Current State of Disease Diagnosis

### 2.1. Defining Normal and Abnormal Levels of Cytokines

It is particularly challenging to evaluate cytokines' diagnostic ability due to the difficulty of establishing “normal” versus “abnormal” cytokine levels. Cytokines vary greatly among individuals, and their release and subsequent effects can differ based upon activating signals, specific cell targets, and physiological factors including stress, fitness level, and feeding state [12]. Cytokines also can vary in different physiological locations and environments, and thus studies that measure cytokines in abnormal and normal circumstances must only compare results with other studies of the same biological fluid (e.g., serum, amniotic fluid, and pleural fluid). Furthermore, only few studies have been conducted to investigate cytokine levels in healthy subjects, and there have been a limited number of variables explored when considering healthy subjects' cytokine profiles [13–15]. Therefore, most studies have established differing “normal” cytokine profiles based on the characteristics of their study populations and the modes of cytokine measurement. The factors are as follows: (a) variation exists in what is considered to be a “normal” cytokine profile; (b) few conclusions have been drawn across studies to define normal cytokine levels; and (c) a variety of factors contribute to cytokine release and action. Hence, these studies often define cytokine levels only within the population of interest. Most studies do not use preestablished cutoffs for cytokine reference values but rather consider the median or mean cytokine levels of healthy subjects in a defined set of population to be “normal.” This mean or median cytokine level for a particular study is then used as a reference cutoff to identify comparatively abnormal cytokine levels in diseased patients; that is, levels are only considered abnormal if they differ from the mean of that population by approximately 2 standard deviations, though this is not diagnostically reliable. Therefore, it is necessary to conduct a greater number of studies within various populations, controlling the aforementioned factors, in order to establish normal cytokine levels and therefore have a more uniform reference for “abnormal” cytokine levels for use in supplementing clinical diagnosis.

A number of bioassay and immunoassay methods are used in clinical practice to detect cytokines currently, though immunoassays are most often used because of their specificity for individual cytokines [16]. Common immunoassay techniques include enzyme-linked immunosorbent assays (ELISA), multiplex arrays, bead-based assays, and the recently studied immunosensing method [12]. Cytokines can also be measured indirectly, using mRNA transcripts, though these are not always an indication of cytokine activity and instead represent the potential for cytokine production. Therefore, direct protein detection uses are most often used for improved interpretation of physiologic cytokine activity. Direct immunodetection assays vary in consistency and reliability, cost, required time, ease of use, throughput, and sample volumes required, among other factors. Currently, the most popular way to detect cytokines is through ELISA, which involves immobilization of proteins and their detection using antibodies, either directly or indirectly [12]. Although this method is commonly used, it is time-consuming and permits analysis of only one cytokine at a time. A method which has improved upon these drawbacks is the multiplex array, a similar protein detection method which permits measurement of multiple cytokines at once [17]. A more recent approach to detecting cytokines is known as immunosensing, which transduces antigen-antibody interactions into electrical signals, though this technique is not yet well studied [18]. As such methods for cytokine detection improve, the complexity of cytokine interactions and effects can be more accurately portrayed.

### 2.2. Establishing Clinical Cutoffs Using Receiver Operating Characteristics Analysis

Due to lack of conclusiveness regarding normal and abnormal cytokine levels, particular clinical cytokine cutoffs for disease states are difficult to establish. In other words, cutoffs for normal and abnormal cytokines in disease states need to be more well-established in order to more accurately support and distinguish between diagnoses, as well as estimate prognoses.

A common way of evaluating diagnostic accuracy of individual biomarkers is by using Receiver Operating Characteristics Analysis (ROC analysis), which plots the true positive rate (“benefits”) versus the false positive rate (“costs”) of a particular disease at different cutoffs of the implicated biomarker [19]. This type of analysis indicates the levels of the biomarker that are most diagnostically useful, allowing the ruling out of disease, through sensitivity values, or allowing disease to be essentially confirmed, through specificity values. An optimal cutoff can be established by identifying the point on the ROC curve with the highest sensitivity and specificity, for maximum diagnostic discriminatory ability [20]. A limited number of studies have utilized ROC analysis to evaluate diagnostic utility of cytokines for particular disease states. The majority of conditions for which cytokine associations and their clinical cutoffs have been explored can be divided into three categories: infections and postoperative infections, inherited and chronic diseases, and obstetric and gynecological conditions. These associations and clinical cutoffs will be discussed below, along with their implications for clinical testing and future work.

## 3. Role of Cytokines and Their Clinical Cutoffs in Infection Diagnosis and Postprocedural Infection Diagnosis

Correlations of abnormal cytokine profiles with infection, including tuberculosis, pneumonia, and systemic inflammatory response state (SIRS), have been investigated in various studies. Hospital-acquired and postoperative infections were also found to have associations with abnormal cytokine profiles, including neonatal sepsis, periprosthetic joint infection, and postcervical neck dissection infection. More importantly, beyond simple associations between abnormal cytokine profiles and these infections, a limited number of studies have also investigated clinical cytokine cutoffs to support diagnosis, which is unprecedented in various other disease states.

### 3.1. Tuberculosis, Pneumonia, and SIRS State

One of the most widely investigated areas of cytokine clinical cutoffs is in the diagnosis of tuberculosis (Tb). In 2003, Wong et al. suggested the ability to diagnose Tb using IL-6, TNF-*α*, and IFN-*γ* in pleural fluid, as patients with Tb pleural effusions are known to have significantly higher pleural effusion cytokine levels than non-Tb patients [21]. The study established cutoffs with considerable predictive and diagnostic value for Tb with pleural effusions: 4000 pg/mL for IL-6 (sensitivity: 90.6%; specificity: 76.5%), 4 pg/mL for TNF-*α* (sensitivity: 90.6%; specificity: 79.4%), and 60 pg/mL for IFN-*γ* (sensitivity: 100%; specificity: 100%). The following year, Sharma and Banga (2004) specifically investigated IFN-*γ* as a predictor of Tb pleural effusions [22]. In this study population, the IFN-*γ* levels of Tb patients were significantly higher than healthy individuals (1480 pg/mL versus 3 pg/mL, resp.), and it was determined that the best cutoff of pleural fluid IFN-*γ* to predict Tb pleural effusion was 138 pg/mL (AUC 95.4%, sensitivity: 90.2%, specificity: 97.3%). The study also examined IFN-*γ* in peritoneal fluid ascites to determine cytokine clinical cutoffs for Tb and discovered an optimal cutoff for IFN-*γ* in peritoneal ascites of 112 pg/mL (AUC 99.0%, sensitivity: 97%, specificity: 97%). However, IFN-*γ* assays using both pleural effusions [22] and peritoneal ascites [23] are considerably expensive, which may render this method less clinically useful and practical. Küpeli et al. (2008) investigated a less expensive method of testing for cytokines as diagnostic biomarkers of Tb, using serum and bronchoalveolar lavage fluid (BALF) [24]. This study found a TNF-*α* cutoff of 17.6 pg/mL in serum and BALF (sensitivity: 73%; specificity: 76%) for distinguishing patients with smear-negative Tb from healthy subjects. However, Küpeli et al. (2008) did not find significant differences between smear-negative Tb patients and non-Tb groups when distinguishing using IL-12 and IFN-*γ* and therefore did not evaluate clinical diagnostic cutoffs for these cytokines [24]. Most recently, Shu et al. (2015) proposed that IFN-*γ* cannot be used alone and demonstrated that, in order to improve the IFN-*γ* model, DcR3 and TNF-sR1 should be included when developing an ROC curve for predicting Tb pleural effusion, suggesting that additional factors must be considered when using cytokines as diagnostic tools [25].

Cytokines were also investigated in ventilator-associated pneumonia (VAP), a disease with similar clinical signs and symptoms to Tb. A 2009 study found that VAP can be predicted and diagnosed using only serum levels of IL-6, as other cytokines did not prove to be associated or predictive of VAP [26]. To distinguish between patients who did and did not subsequently develop VAP, a baseline IL-6 cutoff value of 198 pg/mL was determined (sensitivity: 71%; specificity: 78%). To distinguish patients with confirmed VAP versus suspected VAP, an IL-6 cutoff for the disease state was established to be 620 pg/mL (sensitivity: 71%; specificity: 89%). Morris et al. identified two additional serum cytokines, which can be used to predict VAP, IL-1*β* and IL-8, among others, including IL-6, TNF-*α*, and IL-10 [27]. Clinical cutoff values of IL-1*β* and IL-8 for diagnosing VAP were 10 pg/mL (sensitivity: 94%; specificity: 64%) and 2000 pg/mL (sensitivity: 81%; specificity: 83%), respectively. Although these studies suggest a particular cytokine profile for identifying VAP, it is still necessary to distinguish the condition from Tb due to their similar symptoms and clinical signs. The previously mentioned studies can identify VAP, or Tb, though they cannot necessarily distinguish between the two. Su et al. (2010) investigated cytokines that may be able to distinguish between the two conditions to confirm diagnosis of either Tb or pneumonia, finding IFN-*γ* and IL-12 to yield considerable results through ROC analysis [28]. When cells were stimulated with ESAT-6, a cutoff of a 3.59% change in IFN-*γ* was found to yield sensitivity, specificity, and accuracy of over 80% for diagnosing Tb (90.4% AUC). When diagnosing pneumonia in cells stimulated with LPS, a cutoff of 3.59% change in IFN-*γ* produced sensitivity, specificity, and accuracy of 80% (89.1% AUC), and a cutoff of 2.08% change in IL-12 produced sensitivity of 80%, specificity of 78.9%, and accuracy of 79.4% (85.2% AUC). These results suggest that pneumonia and Tb may be distinguished diagnostically by cytokine responses of IFN-*γ* and IL-12 upon cell stimulation by different reagents and also suggest such laboratory testing to rapidly support diagnosis of these diseases in light of the slow rate at which other clinical features (such as bacterial infection) may be measured and observed.

Another related form of inflammation with respect to cytokines is the limited published work regarding SIRS state. This is an important area of research to explore because systemic inflammation can be caused by a variety of factors, including trauma, and may lead to other previously discussed infections (such as pneumonia). In a distinctive study, Giannoudis et al. (2008) found that IL-6 was predictive of a SIRS state at all points following hospital admittance for trauma (femoral shaft fracture) [29]. At days 0 and 1 after admittance, a cutoff of 20,000 pg/mL IL-6 diagnosed a SIRS state (83% sensitivity; 75% specificity), and an IL-6 level above 300 pg/mL in SIRS patients was correlated with larger risks of complications, including pneumonia and death. Therefore, diagnosing a SIRS state might become more feasible, if more work is done to investigate these cutoffs, which will help prevent further complications that result from such an inflammatory state.

### 3.2. Neonatal Sepsis

A more well-studied phenomenon with respect to cytokine clinical cutoff values is neonatal sepsis, which is a bacterial bloodstream infection that typically appears in infants within twenty-four hours of birth. A litany of studies has connected neonatal sepsis to abnormal cytokine profiles, though only a small number have investigated specific cytokine cutoffs for diagnosing neonatal infection. Yet compared to other conditions, neonatal sepsis has been more thoroughly studied with respect to cytokine cutoffs and thus is an excellent example for the direction in which other studies can be conducted in the future.

Three cytokines, IL-6, TNF-*α*, and IL-1*β*, were investigated and strongly implicated as diagnostic tools in neonatal sepsis [30]. The study established cutoffs facilitating diagnosis on day one of infection: IL-6: 31 pg/mL; TNF-*α*: 17 pg/mL; IL-1*β*: 1 pg/mL. Combination of multiple markers enhanced accuracy of the tests, particularly when IL-6 and TNF-*α* were combined (sensitivity: 95%; specificity: 84%). Timing and duration of infection upon testing seemed to affect diagnostic capabilities of these tests as well: IL-6 appeared to have greater diagnostic value on the day of infection onset (sensitivity: 89%; specificity: 96%), and TNF-*α* had greater diagnostic value on the day after infection onset (sensitivity: 82%; specificity: 93%). Notably, optimal calculated values for clinical cutoffs differed considerably from cutoff values recommended by the manufacturer of the serum test, which suggests a need for future similar work to confirm cytokine cutoffs in establishing detection limits.

Recent studies have also established highly sensitive and specific IL-6 cutoffs for diagnosing neonatal sepsis [31, 32]. It was identified that initial neonatal sepsis onset (within the first 24 hours of infection) was best diagnosed with a combination of IL-6 and CRP detection, with a cutoff of 18 pg/mL for IL-6 and 10 pg/mL for CRP (sensitivity: 89%; specificity: 73%) [31]. However, the same study also found that one day after sepsis onset, CRP alone was a better predictor of infection, warranting future work. Another study, by Sherwin et al. (2008), reported a variety of cytokines using ROC analysis, including Il-1*β*, IL-6, IL-12, IL-8, IL-10, and TNF-*α* [33]. This study found that IL-12 was the most promising source of diagnostic aid, particularly in confirming neonatal sepsis, and that IL-6 was not as effective in predicting the condition. Evaluating use of IL-12 in diagnosis of neonatal sepsis yielded an optimal cutoff of 75 pg/mL (AUC 74%; sensitivity: 28%; specificity: 98%). IL-10 was the next most promising diagnostic aid, with the same cutoff but different test characteristics (sensitivity: 17%; specificity: 99%). In these results, one must consider low sensitivities for these tests, which imply that these cytokine biomarkers can be more accurately used to confirm diagnosis of neonatal sepsis, rather than confirming its absence.

Boskabadi et al. conducted a variety of studies regarding clinical cytokine cutoffs for diagnosing neonatal sepsis: they found IL-8 at a cutoff of 60 pg/mL to be predictive of the disease [34]. In subsequent studies by the same group, IL-10 was reported to have the greatest predictive value at a cutoff of 14 pg/mL [32, 35]. Among IL-6, IL-8, and IL-10, both IL-6 and IL-8 were found to predict neonatal sepsis at cutoffs of 10.85 pg/mL (sensitivity: 92.5%; specificity: 97.6%) and 60.05 pg/mL (sensitivity: 93.7%; specificity: 65%), respectively [32]. IL-6 predicted mortality due to neonatal sepsis, with a higher cutoff of 78.2 pg/mL (sensitivity: 85%; specificity: 76%). As evidenced by these studies, IL-6, IL-8, and IL-10 may be the most promising cytokine biomarkers to diagnose neonatal sepsis once more narrow ranges in cutoffs across studies are established. IL-6 is of special interest, due to its potential ability to both diagnose neonatal sepsis and predict mortality due to the condition, as well as its relatively uniform range in cutoffs across these preliminary studies.

### 3.3. Surgical Site Infections: Periprosthetic Joint Infection and Postcervical Neck Dissection Infection

A variety of other infection pathologies have been connected with cytokines, though very few have been established as predicted by particular cytokine cutoffs. Among these, periprosthetic joint infection (PJI) is a risk after revision surgery for shoulder arthroplasty and is considerably difficult to diagnose due to a lack of biomarkers to distinguish between septic and aseptic outcomes. Cytokines in synovial fluid may be able to aid in diagnosis of PJI, based on correlative studies, though few have evaluated particular cytokine cutoffs for the condition. Frangiamore et al. (2015) found that IL-6 in synovial fluid could help predict PJI after revision surgery for shoulder arthroplasty, with an optimal cutoff of 359.3 pg/mL (sensitivity: 87%; specificity: 90%) [36]. The most recent study of Frangiamore et al. in this area (2016) also investigated IL-1*β* and IFN-*γ* as potential diagnostic biomarkers for the disease, at cutoffs of 8.26 pg/mL (AUC 92% and 91%, resp.) and 34 pg/mL [37]. Perhaps more importantly, IL-1*β* and IL-6 had the highest sensitivities as diagnostic tools (90% and 81%, resp.), and both also had the greatest decrease between explantation and reimplantation demonstrating infection resolution (12.4-fold decrease for IL-1*β*; 11.2-fold decrease for IL-6), implicating IL-1*β* and IL-6 as potentially the most applicable for usage in diagnosis/prognosis of PJI. It is also important to note that all cytokines measured did not show significant diagnostic utility or sensitivity to rule out infection before reimplantation. Cytokine assays have the potential to be significant in PJI diagnosis, as synovial fluid cytokine testing may be more sensitive than current testing available for diagnosing PJI, which may promise better patient outcomes.

In a similar vein, another study demonstrated statistical differences between patients with and without surgical site infection (SSI) after cervical neck dissection on postsurgical drainage fluid cytokine levels, including IL-1*β*, IL-2, IL-6, IL-8, and TNF-*α* [38]. IL-10 was the only cytokine not associated with SSI. TNF-*α* and IL-1*β* showed the greatest diagnostic efficacy of the cytokines associated with SSI: sensitivities and specificities were 100% and 87.88% for TNF-*α* at a cutoff of 14.5 pg/mL (on day 1 of infection for TNF-*α*) and 83.33% and 78.79% for IL-1*β* at a cutoff of 115 pg/mL (on day 3 of infection for IL-1*β*). IL-2 levels were also indicative of disease on days 1 and 3 above levels of 6.5 pg/mL, and IL-6 levels were indicative on day three, above levels of 3,300 pg/mL, though at lower sensitivity and specificity values. This study emphasizes the importance of temporally sensitive evaluation of cytokines, due to their variation in release and concentration over time, along with the promise of potential cytokine biomarkers for infection prediction and diagnosis at various stages of infection.

### 3.4. Recovery from Procedure for Left Ventricular Dysfunction and Prognosis of Heart Failure

Cytokine cutoffs may also be established to evaluate prognosis of particular diseases with inflammatory indications, including left ventricular dysfunction (LVD), treated by percutaneous coronary intervention (PCI) and its often associated subsequent heart failure, or acute myocardial infarction (AMI). Left ventricular dysfunction is a risk factor for poor prognosis following acute myocardial infarction (AMI) [39], which has been associated with an imbalance in pro- and anti-inflammatory markers, and therefore some studies have sought to investigate potential correlations between these conditions and cytokines for improved prognosis evaluation. Szkodzinski et al. (2011) investigated cytokines predicting LVD before and after PCI to evaluate possible cytokine predictors [39]. Measurements of serum cytokines before and after PCI showed high diagnostic value of IL-4 both before and immediately after PCI, whereas IFN-*γ* measurements only before PCI were of diagnostic aid. IL-4 at levels above 15 pg/mL before PCI indicated LVD, and IL-4 at slightly higher levels of 17.2 pg/mL after PCI indicated LVD. IFN-*γ* predicted LVD before PCI with a cutoff of 0.3 pg/mL. Caruso et al. (2014) attempted to further this evaluation of LVD, identifying potential cytokine cutoffs to predict prognosis of LVD patients in those who require left ventricular assist devices (LVAD) [40]. Among LVD patients, many require a LVAD to aid in preventing the development of multiorgan failure (MOF). Aside from the inflammatory indications of LVD, unique inflammatory profiles also appear upon implantation of the assist device. In this study, white blood cell count, indicating infection, and MOF severity were associated with IL-6 at levels above 8.3 pg/mL. This cutoff of IL-6 also indicated longer ICU stay, longer hospitalization, poor early outcome, and higher levels of release of other proinflammatory molecules, such as IL-8, which can help evaluate prognosis of patients with LVAD long-term. These studies indicate the potential for particular interleukins to aid in diagnosis of LVD, regardless of surgical intervention, and in prognosis of LVD, in patients with LVAD.

## 4. Role of Cytokines and Their Clinical Cutoffs in Evaluation of Severity and Prognosis for Chronic Conditions

Aside from infections and diseases with inflammatory indications, cytokines may also be useful in assessing prognoses of inherited and often chronic conditions, including Alzheimer's Disease, neurological outcomes following cardiac arrest (causing prolonged hypoxia), cancer, lupus nephritis, and lymphohistiocytosis. Conditions such as these have been correlated with elevated proinflammatory cytokine levels in previous work, though few have investigated particular cytokine cutoffs for predicting severity and prognosis of these diseases.

### 4.1. Alzheimer's Disease and Neurological Outcomes in Cardiac Arrest Patients

Alzheimer's Disease (AD) has been well established as a disease which is caused by inflammatory processes, and cytokines have accordingly been implicated in its etiology. However, cutoffs of cytokines in diagnosing AD have not been well studied. A recent study in a Malaysian population demonstrated the possibility of establishing a particular cutoff for cytokines and chemokines in AD [41]. In the blood, IP-10 (a chemokine) and IL-13 were both associated with AD: IL-13 was 18-fold lower in AD patients than in healthy controls, and IL-13 was 9-fold lower in European AD patients when compared to healthy controls. Another study also found that anti-inflammatory IL-13, as well as IP-10, was downregulated in AD patients compared to controls [42]. An IL-13 cutoff value of 9.315 pg/mL was determined (sensitivity and specificity 100%) to diagnose AD, in accordance with the anti-inflammatory properties of IL-13. The suggestion of such a specific and sensitive test for AD using cytokines is significant to the diagnostic field of this disease: standard methods for diagnosing Alzheimer's are costly, as they require highly trained specialist physicians and expensive imaging methods. Further, a method that relies purely on serum measurements of cytokines would facilitate diagnosis of AD, particularly in poorer nations.

In addition to poor neurological outcomes resulting from AD, such outcomes can also result from cardiac arrest due to prolonged oxygen deprivation, and these effects may be identified by cytokine cutoffs. Oda et al. (2009) measured cytokines in cerebrospinal fluid (CSF) in patients 6 months following cardiac arrest and evaluated patients according to the Glasgow Outcome Score (GOS) in order to evaluate poor neurological outcome as indicated by CSF cytokines [43]. IL-8 and IL-6 were found to be significantly higher in subjects who had experienced cardiac arrest, and both cytokines were found to be correlated with poor neurological outcome. Cytokine cutoffs for predicting poor neurological outcome following cardiac arrest were 1423 pg/mL for IL-8 and 2708 pg/mL for IL-6 (both with sensitivity of 100% and specificity of 86%). These studies suggest that, following future work, evaluation of poor neurological prognosis may be possible in cases of both AD and cardiac arrest through serum and CSF cytokine levels, respectively.

### 4.2. Gastric Cancer

Gastric cancer is another condition characterized by an abnormal cytokine profile. Various studies have linked IL-6 to gastric cancer, though few studies have investigated clinical cutoffs of IL-6 for this condition. Most studies of cytokines in cancer, as in diagnosis of other conditions using cytokines, set a cutoff of a particular percentile rather than considering ROC analysis for diagnostic efficacy. However, despite considerable precedent, a 2005 study found the optimal diagnostic cutoff of IL-6 for gastric cancer to be 1.97 pg/mL (sensitivity 81.8%; specificity: 66.7%) [44]. This corresponded with an accuracy of 77.1% as well as significantly lower patient survival rates and positive immunohistochemical staining of IL-6 in cancer cells. A slightly higher serum cutoff of this cytokine was identified as having the potential to diagnose preoperative gastric cancer and to evaluate prognoses after surgical tumor removal: 6.77 pg/mL serum IL-6 (sensitivity 85.7%) [45]. However, the consensus between the two studies regarding the role of IL-6 in predicting gastric cancer suggests that the correct cytokine for diagnostic use has been identified and that it will just be a matter of time to establish a narrower diagnostic cutoff range with improved sensitivity.

### 4.3. Lupus Nephritis and Lymphohistiocytosis

Lupus nephritis and lymphohistiocytosis are chronic conditions of immune deficiency. Lupus nephritis often accompanies systemic lupus erythematous (SLE) and is characterized by inflammation of the kidney. Lupus nephritis has been associated with immune markers, including cytokines, though few studies have established specific clinical cutoffs for these markers. Torabinejad et al. (2012) investigated TGF-*β*, among other noncytokine immune molecules, and evaluated its effect on diagnosis of lupus nephritis in patients with SLE; the study established a cutoff point of 54.2 pg/mL TGF-*β* (sensitivity: 71.4%; specificity: 95.6%) [46]. Despite low sensitivity, the test was highly specific and can therefore be important in obviating the need for additional diagnostic testing, such as renal biopsies for certain patients. If further studies are conducted to establish a cutoff with higher sensitivity, this cytokine may be used diagnostically. A 2015 study determined cutoffs of cytokines IL-17 and IL-6 to diagnose lupus nephritis in patients with SLE [47]. Activity of these cytokines was present at a significant level, both during active disease periods and during remission. Optimal cutoffs for diagnosing active forms of lupus nephritis using IL-6 and IL-17 were 12.3 pg/mL (AUC 93%) and 19.7 pg/mL (AUC 95%), respectively, whereas optimal cutoffs for diagnosing lupus nephritis in remission were higher, at 20.8 pg/mL (AUC 80%) and 27 pg/mL (AUC 82%), respectively. Accordingly, these biomarkers may be used not only to diagnose lupus nephritis, but also to predict remission of the disease.

Hemophagocytic lymphohistiocytosis (HLH) is an inherited immune deficiency resulting from dysfunction of T and natural killer cells. Xu et al. (2012) considered IFN-*γ*, TNF-*α*, IL-10, IL-6, IL-4, and IL-2 as potential biomarkers of this disease in pediatric febrile patients [48]. In patients with HLH, the median level of IFN-*γ* was 1088.5 pg/mL, and the median level of IL-10 was 623.5 pg/mL. IL-6 was elevated to a lesser extent, with a median level in these HLH patients of 51.1 pg/mL, though among HLH patients with sepsis, as expected, a higher median IL-6 level was detected (244.6 pg/mL). Upon ROC analysis, IFN-*γ* had a sensitivity of 94.4% and specificity of 97.2% for diagnosing HLH at a 100 pg/mL cutoff. IL-10 did not provide considerable diagnostic utility individually, but when IFN-*γ* and IL-10 were considered at cutoffs of >75 pg/mL and >60 pg/mL, respectively, specificity increased to 98.9% and the sensitivity increased to 93%. However, IL-6, though it moderately increased in these patients, did not appear to be diagnostically useful. Despite its lack of diagnostic relevance, IL-6 levels may help contribute to the characterization of the profile of pediatric febrile HLH patients in its moderate level, among higher levels of IFN-*γ* and IL-10. It will become necessary to consider nonfebrile patients in future studies due to the possibility of the fever state contributing to the inflammatory process, though this study and the previous studies of lupus nephritis may be useful at gaining a preliminary understanding of the particular levels of cytokines at which the body responds to immune deficiencies.

## 5. Role of Cytokines and Their Clinical Cutoffs in Obstetric and Gynecological Conditions

The third category of conditions where cytokines can be evaluated as biomarkers is obstetric and gynecological conditions characterized by inflammation, including endometriosis, ovarian abnormalities, ectopic pregnancy, miscarriage, preterm premature rupture of membranes (PROM), and preterm delivery. Such conditions have been significantly correlated with cytokines in various studies, though recently, and perhaps more importantly, clinical cutoffs are being established for diagnosis as well.

### 5.1. Endometriosis and Ovarian Cancer

Endometriosis is a condition characterized by a variety of inflammatory biomarkers, though diagnosis typically involves a pelvic exam or imaging. Cytokines have the potential to aid in diagnosis of this condition, reducing the need for costly imaging techniques or uncomfortable physical examinations. Of a number of proinflammatory cytokines, two have been established as potentially useful for diagnostics, IL-6 and TNF-*α*, which were found to be correlated with endometriosis and to be effective in diagnosing endometriosis in serum and peritoneal fluid as well as in menstrual effluents. Bedaiwy et al. (2002) found IL-6 and TNF-*α* to be able to distinguish between patients with and without endometriosis with high sensitivity and specificity, at respective cutoffs of 2 pg/mL (sensitivity 90%; specificity 67%) and 15 pg/mL (sensitivity 100%; specificity 89%) [49]. Tortorella et al. (2014) found significantly higher levels of IL-6, IL-1*β*, and TNF-*α* in menstrual effluents of women diagnosed with endometriosis compared to healthy subjects, though they found limited diagnostic capability of each of these cytokines individually [50]. However, when combined (IL-6 combined with TNF-*α* or IL-6 combined with IL-1*β*), the cytokines provided diagnostic sensitivity of nearly 100% when at least one cytokine was above the diagnostic value and provided a near 100% specificity when both cytokines in a combination exceeded their cutoff values: IL-6, 8,968 pg/mL; TNF-*α*, 203 pg/mL. Logistic regressions demonstrated the combination of IL-6 and TNF-*α* to be significantly predictive of chronic endometriosis diagnosis, confirming their use as diagnostic tools, as suggested by ROC analysis. It is important to note, however, that the IL-6 and TNF-*α* cutoffs differ greatly between the two studies, warranting future work to narrow this range of diagnostic cutoffs. More studies in this area have the potential to avoid unnecessary invasive diagnostic procedures for endometriosis by providing the capacity for a test of either serum, peritoneal fluid, or menstrual effluent, to diagnose or eliminate the probability of disease.

Ovarian cancer has also been associated with a variety of cytokines, though it has only been investigated with respect to diagnostic utility. Chechlinska et al. (2007) measured a number of cytokines (VEGF, IL-6, bFGF, IL-8, and M-CSF) in peritoneal fluid of untreated ovarian cancer patients and those with benign ovarian tumors [51]. Although IL-6, VEGF, and CA 125 all were elevated in ovarian cancer patients, and bFGF and M-CSF were decreased in these patients, only IL-6 and VEGF were significantly elevated in stagees I and II cancer patients. For these two significantly associated cytokines, two cutoff levels were tested: 400 pg/mL and 1200 pg/mL. At these two cutoffs, IL-6 had a sensitivity of 92% and 84%, respectively, and a specificity of 60% and 87%, respectively. At a cutoff of 400 pg/mL, VEGF had a sensitivity of 90% and a specificity of 80%. Accordingly, measurements of these two cytokines in peritoneal fluids may be useful for identifying malignant versus benign ovarian tumors upon first diagnosis. Gorelik et al. (2005) considered a wider range of cytokines in serum, including IL-6, IL-8, EGF, VEGF, MCP-1, and cancer antigen-125, due to evaluated significance in correlations [52]. These cytokines were analyzed through classification tree analysis to determine their optimal combinations to diagnose ovarian cancer. A tree combining all of the aforementioned cytokines, excepting MCP-1, was generated to diagnose ovarian cancer versus controls with sensitivity 84% and specificity 95%; a tree combining CA-125, G-CSF, IL-6, EGF, and VEGF was generated to distinguish ovarian cancer from benign ovarian tumors with sensitivity 86.5% and specificity 93%. Both of these studies suggest IL-6 and VEGF as possible diagnostic biomarkers for ovarian cancer with reasonable sensitivities and specificities for clinical applications.

### 5.2. Ectopic Pregnancy and Miscarriage

Ectopic pregnancy has been correlated with proinflammatory cytokines, including IL-2R, IL-6, IL-8, and TNF-*α*, and these cytokines have been investigated as diagnostic biomarkers with specific cutoffs. These four cytokines were measured in serum levels of women with miscarriage, normal intrauterine pregnancy, and ectopic pregnancy (EP). One study found that higher IL-8, IL-6, and TNF-*α* were present in women with EP when compared to women with miscarriage and normal pregnancy, though only IL-8 yielded a cutoff, of 40 pg/mL, for predicting ectopic pregnancy with notable sensitivity and specificity (82.4% and 81.8%, resp.) [53]. Another study found potential utility of serum IL-6 and IL-8 in diagnosing ectopic pregnancy, as compared to normal intrauterine pregnancy and miscarriage [54]. However, this study only found IL-6 to yield remarkable sensitivity and specificity for ectopic pregnancy diagnosis at a cutoff of 26.48 pg/mL and did not find IL-8 to be significantly predictive of the condition at any cutoff. Based on these findings, it is possible that within a larger study, IL-6, IL-8, or a combination of the two may be determined useful as noninvasive markers for predicting ectopic pregnancy.

### 5.3. Preterm PROM and Preterm Delivery

Preterm PROM is characterized by rupture of the amniotic sac before the fetus is carried to term and is often associated with inflammation due to microbial invasion, as indicated by amniotic fluid cytokine levels obtained through amniocentesis. The associated inflammation and microbial invasion in this condition have been most conclusively correlated with proinflammatory IL-6 in amniotic fluid, though the specific cutoffs of this biomarker suggest the disease have only been recently and preliminarily investigated. Kacerovsky et al. (2014) sought to evaluate the diagnostic utility of IL-6 in diagnosing microbial invasion of the amniotic cavity in PROM pregnancies; a cutoff of 1000 pg/mL IL-6 in amniotic fluid was determined to be the optimal diagnostic level for identifying microbial amniotic invasion (sensitivity 50%, specificity 95%) [55]. The study also identified a 1000 pg/mL cutoff in PROM patients for identifying histological chorioamnionitis in combination with microbial amniotic invasion (sensitivity 60%, specificity 94%). However, amniocentesis is extremely invasive, prompting Musilova et al. (2016) to test cytokine levels in vaginal fluid of pregnant women in an attempt to predict preterm PROM-related inflammation in a less invasive manner [56]. IL-6 was determined to be associated with microbial invasion of the amniotic cavity, as well as intra-amniotic inflammation and microbial-associated intra-amniotic inflammation, and was able to predict these conditions at a cutoff of 2500 pg/mL with high sensitivity and specificity: 53% and 89%, 74% and 91%, and 100% and 90%, respectively. These consistently higher specificity values suggest the ability of IL-6 to distinguish between pregnant women with and without PROM-related inflammation.

Like preterm PROM, preterm delivery has also been linked to IL-6, though preterm delivery has additionally been correlated with IL-8. These cytokines have primarily been measured in cervicovaginal fluid to evaluate their diagnostic utility due to ease of access and limited cost. Woodworth et al. (2007) investigated IL-6 in cervicovaginal fluid to predict early delivery, finding a 35% sensitivity value and 91% specificity value at a 250 pg/mL [57]. Odds ratios supported this finding, testing IL-6 as a predictor of preterm delivery, with statistical significance (*p* = 0.0001). The IL-6 : albumin ratio provided significance on a lower confidence interval and did not yield a similarly high sensitivity or specificity. Nonetheless, IL-6 levels are suggested to be predictive of preterm delivery within 14 days as an inexpensive and limitedly invasive diagnostic method. Gandevani et al. (2011) also found IL-6 to predict preterm delivery, though Gandevani et al. found IL-8 to be predictive of this condition [58]. Fourfold and nearly fivefold increases in IL-6 and IL-8 concentrations were found in preterm versus normal deliveries, respectively. Cutoffs of these cytokines for predicting early preterm delivery were determined as 751.25 pg/mL IL-8 and 157 pg/mL IL-6 (sensitivities of 89% and specificities of 83% and 78%, resp.). Likewise, Shahshahan and Hashemi (2014) found results suggesting IL-6 and IL-8 to be diagnostic markers for preterm labor and delivery [59]. However, Shahshahan and Hashemi (2014) measured the two cytokines in serum of pregnant women with and without preterm labor, rather than in cervicovaginal fluid, finding IL-6 to have a lower optimal cutoff for predicting preterm labor of 37.8 pg/mL and IL-8 to have a lower optimal cutoff of 9.5 pg/mL. Cytokine levels were also evaluated to predict response to tocolytic therapy in pregnant women with preterm delivery, with cutoffs of 45 pg/mL for IL-6 and 171 pg/mL for IL-8. These studies suggest the possibility of both IL-6 and IL-8 as predictors of preterm delivery and the response of such pregnancies to tocolytic therapy, though further work must be conducted to narrow the range of cutoffs for diagnostic utility.

## 6. Conclusion

### 6.1. Future of Cytokine Research in the Diagnostic Field

Many of the studies mentioned above are unprecedented in their search for cytokine cutoffs to aid in diagnosis of particular conditions, as many studies simply search for correlations between cytokines and disease states without evaluating specific clinical cutoffs. Although cytokines are implicated in various disease states and are dependent upon a variety of pathways, studies such as those reported in this review demonstrate the possibility of using cytokines or combinations of cytokines, in addition to other factors, to diagnose various immunologically implicated conditions with greater ease and accuracy, and at a lower cost.

The establishment of clinical cytokine cutoffs for various conditions may be facilitated by future improvement in our understanding of normal cytokine profiles. To better understand cytokine levels and to establish diagnostic cutoffs in disease states, it is important to first characterize normal cytokine levels in various populations and to understand how cytokines interact and modulate in various biochemical pathways in healthy individuals. Particularly, if lack of research in certain diseases' areas prevents the use of cytokines alone in diagnosis, gaining a better understanding of normal cytokine profiles will also allow confirmation and supplementation or reconsideration, of diagnoses made through other diagnostic methods and clinical features. Further, as normal and diagnostic cutoffs for these biomarkers become elucidated, cytokines may be considered to improve patient outcomes when definitive diagnosis is not possible with clinical features alone. Many diseases have very similar clinical features, and therefore establishment of pathological cytokine profiles may aid in differentiation of such conditions.

Due to the limited number of studies investigating effectiveness of particular cytokine cutoffs as diagnostic tools, further studies must be conducted to narrow diagnostic ranges. Such a narrowing of diagnostic ranges will facilitate confirmation or rejection of diagnoses suggested by other clinical features to ultimately improve patient outcomes.

## References

[1] Dinarello C. (2007). Historical insights into cytokines. *European Journal of Immunology*.

[2] Su D.-L., Lu Z.-M., Shen M.-N., Li X., Sun L.-Y. (2012). Roles of pro- and anti-inflammatory cytokines in the pathogenesis of SLE. *Journal of Biomedicine and Biotechnology*.

[3] Pinto L. M. O., Oliveira S. A., Braga E. L. A., Nogueira R. M. R., Kubelka C. F. (1999). Increased pro-inflammatory cytokines (TNF-*α* and IL-6) and anti-inflammatory compounds (sTNFRp55 and sTNFRp75) in Brazilian patients during exanthematic dengue fever. *Memorias do Instituto Oswaldo Cruz*.

[4] Marie C., Pitton C., Fitting C., Cavaillon J.-M. (1996). Regulation by anti-inflammatory cytokines (IL-4, IL-10, IL-13, TGF*β*) of interleukin-8 production by LPS- and/or TNF*α*-activated human polymorphonuclear cells. *Mediators of Inflammation*.

[5] Cavaillon J. M. (2001). Pro- versus anti-inflammatory cytokines: myth or reality. *Cellular and Molecular Biology*.

[6] Williams I. R., Rich B. E., Kupper T. S., Wolff K., Goldsmith L. A., Katz S. I., Gilchrest B. A., Paller A. S., Leffel D. J. (2008). Cytokines. *Fitzpatrick's Dermatology in General Medicine*.

[7] Heinrich P. C., Behrmann I., Müller-Newen G., Schaper F., Graeve L. (1998). Interleukin-6-type cytokine signalling through the gp130/Jak/STAT pathway. *Biochemical Journal*.

[8] Hayden M. S., Ghosh S. (2008). Shared principles in NF-*κ*B signaling. *Cell*.

[9] Conti P., Kempuraj D., Kandere K. (2003). IL-10, an inflammatory/inhibitory cytokine, but not always. *Immunology Letters*.

[10] Scheller J., Chalaris A., Schmidt-Arras D., Rose-John S. (2011). The pro- and anti-inflammatory properties of the cytokine interleukin-6. *Biochimica et Biophysica Acta*.

[11] Mosser D. M., Zhang X. (2008). Interleukin-10: new perspectives on an old cytokine. *Immunological Reviews*.

[12] Zhou X., Fragala M. S., McElhaney J. E., Kuchel G. A. (2010). Conceptual and methodological issues relevant to cytokine and inflammatory marker measurements in clinical research. *Current Opinion in Clinical Nutrition and Metabolic Care*.

[13] Kim H. O., Kim H.-S., Youn J.-C., Shin E.-C., Park S. (2011). Serum cytokine profiles in healthy young and elderly population assessed using multiplexed bead-based immunoassays. *Journal of Translational Medicine*.

[14] Kleiner G., Marcuzzi A., Zanin V., Monasta L., Zauli G. (2013). Cytokine levels in the serum of healthy subjects. *Mediators of Inflammation*.

[15] Chapman P., Reyes C., Gupta V. (2010). Normal physiological levels of human cytokines using Bio-Plex Pro Cytokine Assays. *Bio-Rad Bulletin: Technical Note*.

[16] Bienvenu J., Monneret G., Fabien N., Revillard J. P. (2000). The clinical usefulness of the measurement of cytokines. *Clinical Chemistry and Laboratory Medicine*.

[17] Leng S. X., McElhaney J. E., Walston J. D., Xie D., Fedarko N. S., Kuchel G. A. (2008). ELISA and multiplex technologies for cytokine measurement in inflammation and aging research. *Journals of Gerontology. Series A Biological Sciences and Medical Sciences*.

[18] Liu G., Qi M., Hutchinson M. R., Yang G., Goldys E. M. (2016). Recent advances in cytokine detection by immunosensing. *Biosensors and Bioelectronics*.

[19] Fawcett T. (2006). An introduction to ROC analysis. *Pattern Recognition Letters*.

[20] Fan J., Upadhye S., Worster A. (2006). Understanding receiver operating characteristic (ROC) curves. *Canadian Journal of Emergency Medicine*.

[21] Wong C.-F., Yew W.-W., Leung S. K.-F. (2003). Assay of pleural fluid interleukin-6, tumuor necrosis factor-alpha and interferon-gamma in the diagnosis and outcome correlation of tuberculous effusion. *Respiratory Medicine*.

[22] Sharma S. K., Banga A. (2004). Diagnostic utility of pleural fluid IFN-*γ* in tuberculosis pleural effusion. *Journal of Interferon and Cytokine Research*.

[23] Sharma S. K., Tahir M., Mohan A., Smith-Rohrberg D., Mishra H. K., Pandey R. M. (2006). Diagnostic accuracy of ascitic fluid IFN-*γ* and adenosine deaminase assays in the diagnosis of tuberculous ascites. *Journal of Interferon and Cytokine Research*.

[24] Küpeli E., Karnak D., Beder S., Kayacan O., Tutkak H. (2008). Diagnostic accuracy of cytokine levels (TNF-*α*, IL-2 and IFN-*γ*) in bronchoalveolar lavage fluid of smear-negative pulmonary tuberculosis patients. *Respiration*.

[25] Shu C.-C., Wang J.-Y., Hsu C.-L. (2015). Diagnostic role of inflammatory and anti-inflammatory cytokines and effector molecules of cytotoxic T lymphocytes in tuberculous pleural effusion. *Respirology*.

[26] Ramírez P., Ferrer M., Gimeno R. (2009). Systemic inflammatory response and increased risk for ventilator-associated pneumonia: a preliminary study. *Critical Care Medicine*.

[27] Morris A. C., Kefala K., Wilkinson T. S. (2010). Diagnostic importance of pulmonary interleukin-1*β* and interleukin-8 in ventilator-associated pneumonia. *Thorax*.

[28] Su W.-L., Perng W.-C., Huang C.-H. (2010). Identification of cytokines in whole blood for differential diagnosis of tuberculosis versus pneumonia. *Clinical and Vaccine Immunology*.

[29] Giannoudis P. V., Harwood P. J., Loughenbury P., Van Griensven M., Krettek C., Pape H.-C. (2008). Correlation between IL-6 levels and the systemic inflammatory response score: can an IL-6 cutoff predict a SIRS state?. *The Journal of Trauma*.

[30] Ng P. C., Cheng S. H., Chui K. M. (1997). Diagnosis of late onset neonatal sepsis with cytokines, adhesion molecule, and C-reactive protein in preterm very low birthweight infants. *Archives of Disease in Childhood: Fetal and Neonatal Edition*.

[31] Laborada G., Rego M., Jain A. (2003). Diagnostic value of cytokines and c-reactive protein in the first 24 hours of neonatal sepsis. *American Journal of Perinatology*.

[32] Boskabadi H., Maamouri G., Afshari J. T. (2013). Evaluation of serum interleukins-6, 8 and 10 levels as diagnostic markers of neonatal infection and possibility of mortality. *Iranian Journal of Basic Medical Sciences*.

[33] Sherwin C., Broadbent R., Young S. (2008). Utility of interleukin-12 and interleukin-10 in comparison with other cytokines and acute-phase reactants in the diagnosis of neonatal sepsis. *American Journal of Perinatology*.

[34] Boskabadi H., Maamouri G., Afshari J. T., Ghayour-Mobarhan M., Shakeri M.-T. (2010). Serum interleukin 8 level as a diagnostic marker in late neonatal sepsis. *Iranian Journal of Pediatrics*.

[35] Boskabadi H., Maamouri G. A., Ghayour-Mobarhan M., Tavakkol Afshari J., Shakeri M. T., Ferns G. A. A. (2011). Early diagnosis of late neonatal sepsis by measuring interleukin 10: a case control study. *Journal of Neonatology*.

[36] Frangiamore S. J., Saleh A., Kovac M. F. (2015). Synovial fluid interleukin-6 as a predictor of periprosthetic shoulder infection. *The Journal of Bone & Joint Surgery—American Volume*.

[37] Frangiamore S. J., Siqueira M. P., Saleh A., Daly T., Higuera C. A., Barsoum W. K. (2016). Synovial cytokines and the MSIS criteria are not useful for determining infection resolution after periprosthetic joint infection explantation. *Clinical Orthopaedics and Related Research*.

[38] Candau-Alvarez A., Gil-Campos M., De La Torre-Aguilar M. J., Llorente-Cantarero F., Lopez-Miranda J., Perez-Navero J. L. (2015). Early modification in drainage of interleukin-1*β* and tumor necrosis factor-*α* best predicts surgical-site infection after cervical neck dissection for oral cancer. *Journal of Oral and Maxillofacial Surgery*.

[39] Szkodzinski J., Hudzik B., Osuch M. (2011). Serum concentrations of interleukin-4 and interferon-gamma in relation to severe left ventricular dysfunction in patients with acute myocardial infarction undergoing percutaneous coronary intervention. *Heart and Vessels*.

[40] Caruso R., Botta L., Verde A. (2014). Relationship between pre-implant interleukin-6 levels, inflammatory response, and early outcome in patients supported by left ventricular assist device: a prospective study. *PLoS ONE*.

[41] Dayana S. M., Lim S. M., Tan M. P. (2014). 115 ∗ IP-10 and IL-13 as potentially new, non-classical blood-based cytokine biomarkers for Alzheimer's disease. *Age and Ageing*.

[42] Mohd Hasni D. S., Lim S. M., Chin A. V. (2016). Peripheral cytokines, C-X-C motif ligand10 and interleukin-13, are associated with Malaysian Alzheimer's disease. *Geriatrics and Gerontology International*.

[43] Oda Y., Tsuruta R., Kasaoka S., Inoue T., Maekawa T. (2009). The cutoff values of intrathecal interleukin 8 and 6 for predicting the neurological outcome in cardiac arrest victims. *Resuscitation*.

[44] Ashizawa T., Okada R., Suzuki Y. (2005). Clinical significance of interleukin-6 (IL-6) in the spread of gastric cancer: role of IL-6 as a prognostic factor. *Gastric Cancer*.

[45] Kim D.-K., Oh S. Y., Kwon H.-C. (2009). Clinical significances of preoperative serum interleukin-6 and C-reactive protein level in operable gastric cancer. *BMC Cancer*.

[46] Torabinejad S., Mardani R., Habibagahi Z. (2012). Urinary monocyte chemotactic protein-1 and transforming growth factor-*β* in systemic lupus erythematosus. *Indian Journal of Nephrology*.

[47] Abdel Galil S. M., Ezzeldin N., El-Boshy M. E. (2015). The role of serum IL-17 and IL-6 as biomarkers of disease activity and predictors of remission in patients with lupus nephritis. *Cytokine*.

[48] Xu X., Tang Y., Song H., Yang S., Xu W., Zhao N. (2012). Original article: diagnostic accuracy of a specific cytokine pattern in hemophagocytic lymphohistiocytosis in children. *The Journal of Pediatrics*.

[49] Bedaiwy M. A., Falcone T., Sharma R. K. (2002). Prediction of endometriosis with serum and peritoneal fluid markers: a prospective controlled trial. *Human Reproduction*.

[50] Tortorella C., Piazzolla G., Matteo M. (2014). Interleukin-6, interleukin-1*β*, and tumor necrosis factor *α* in menstrual effluents as biomarkers of chronic endometritis. *Fertility and Sterility*.

[51] Chechlinska M., Kaminska J., Markowska J., Kramar A., Steffen J. (2007). Peritoneal fluid cytokines and the differential diagnosis of benign and malignant ovarian tumors and residual/recurrent disease examination. *International Journal of Biological Markers*.

[52] Gorelik E., Landsittel D. P., Marrangoni A. M. (2005). Multiplexed immunobead-based cytokine profiling for early detection of ovarian cancer. *Cancer Epidemiology Biomarkers and Prevention*.

[53] Soriano D., Hugol D., Quang N. T., Darai E. (2003). Serum concentrations of interleukin-2R (IL-2R), IL-6, IL-8, and tumor necrosis factor alpha in patients with ectopic pregnancy. *Fertility and Sterility*.

[54] Rajendiran S., Senthil Kumar G. P., Nimesh A., Dhiman P., Shivaraman K., Soundararaghavan S. (2016). Diagnostic significance of IL-6 and IL-8 in tubal ectopic pregnancy. *Journal of Obstetrics and Gynaecology*.

[55] Kacerovsky M., Musilova I., Hornychova H. (2014). Bedside assessment of amniotic fluid interleukin-6 in preterm prelabor rupture of membranes. *American Journal of Obstetrics and Gynecology*.

[56] Musilova I., Bestvina T., Hudeckova M. (2016). Vaginal fluid IL-6 concentrations as a point-of-care test is of value in women with preterm PROM. *American Journal of Obstetrics and Gynecology*.

[57] Woodworth A., Moore J., G'Sell C. (2007). Diagnostic accuracy of cervicovaginal interleukin-6 and interleukin-6:albumin ratio as markers of preterm delivery. *Clinical Chemistry*.

[58] Gandevani S. B., Garshasbi A., Faghih-Zadeh S., Ghazanfari T. (2011). Te value of interleukin-8 and interleukin-6 in cervical secretions as predictors of preterm delivery. *Iranian Journal of Pathology*.

[59] Shahshahan Z., Hashemi L. (2014). Maternal serum cytokines in the prediction of preterm labor and response to tocolytic therapy in preterm labor women. *Advanced Biomedical Research*.

